# CAREGIVER CONVERSATIONS WITH PERSONS WITH MILD COGNITIVE IMPAIRMENT, ALZHEIMER’S DISEASE, AND PARKINSON’S DISEASE

**DOI:** 10.1093/geroni/igae098.3330

**Published:** 2024-12-31

**Authors:** Marie Savundranayagam, Annette Schumann, Joseph Orange, Sharon Pritchard, M Kathleen Pichora-Fuller, Walter Wittich, Paul Mick, Jennifer Campos

**Affiliations:** Western University, London, Ontario, Canada; Western University, London, Ontario, Canada; Western University, London, Ontario, Canada; Western University, London, Ontario, Canada; University of Toronto, Toronto, Ontario, Canada; University of Montreal, Montreal, Quebec, Canada; University of Saskatchewan, Saskatoon, Saskatchewan, Canada; The KITE Research Institute, University Health Network, Toronto, Ontario, Canada

## Abstract

This study describes conversations between persons living with mild cognitive impairment (MCI), Alzheimer’s Disease (AD), Parkinson’s Disease (PD) and their family caregivers, and the possible contributions of sensory loss. Conversations (N=130) between a participant (60% male) and a family caregiver (75% female; spouses) in the COMPASS-ND study were analyzed. Conversations were transcribed verbatim, segmented into communication units (c-units), and analyzed using the trouble-source repair (TSR) paradigm that includes analyses of communication breakdowns, repair initiators, and repair strategies. A one-way analysis of variance was conducted to evaluate between-group differences among sensory and communication variables. The AD group had poorer hearing than the MCI and PD groups. Both AD and MCI groups had poorer vision than the PD group. The MCI group used significantly more c-units than the AD and PD groups. The mean proportion of the TSR sequences was 13% across all groups, but with significantly more sequences in the AD and PD groups than the MCI group. Notably, the proportion of trouble sources created by caregivers was significantly greater for conversations in the AD group than the MCI or PD groups. The proportion of repair initiators used was significantly greater for the AD group than the MCI or PD groups. The intersection of cognition and sensory variables will be illustrated using three case studies. The findings highlight the active participation of persons living with AD and sensory loss in signalling misunderstandings created by caregivers. However, the interface between cognitive and sensory abilities influences the communication among both conversational partners.

